# Dehydropeptidase 1 promotes metastasis through regulation of E-cadherin expression in colon cancer

**DOI:** 10.18632/oncotarget.7033

**Published:** 2016-01-27

**Authors:** Sang Yoon Park, Seon-Jin Lee, Hee Jun Cho, Tae Woo Kim, Jong-Tae Kim, Jae Wha Kim, Chul-Ho Lee, Bo-Yeon Kim, Young Il Yeom, Jong-Seok Lim, Younghee Lee, Hee Gu Lee

**Affiliations:** ^1^ Genome Structure Research Center, Korea Research Institute of Biosceience and Biotechnology, Daejeon, Korea; ^2^ Department of Biomolecular Science, University of Science and Technology (UST), Daejeon, Korea; ^3^ Biomedical Translational Research Center, Korea Research Institute of Biosceience and Biotechnology, Daejeon, Korea; ^4^ Animal Resource Center, Korea Research Institute of Biosceience and Biotechnology, Daejeon, Korea; ^5^ World Class Institute, Korea Research Institute of Bioscience and Biotechnology, Cheongju, Korea; ^6^ Department of Functional Genomics, Korea Research Institute of Biosceience and Biotechnology, Daejeon, Korea; ^7^ Department of Biological Sciences, and The Research Center for Women's Diseases, Sookmyung Women's University, Seoul, Korea; ^8^ Department of Biochemistry, College of Natural Sciences, Chungbuk National University, Cheongju, Korea

**Keywords:** colon cancer, invasion, metastasis, E-cadherin, dehydropeptidase 1

## Abstract

Dehydropeptidase 1 (DPEP1) is a zinc-dependent metalloproteinase that is expressed aberrantly in several cancers. The role of DPEP1 in cancer remain controversial. In this study, we demonstrate that DPEP1 functions as a positive regulator for colon cancer cell metastasis. The expression of DPEP1 mRNA and proteins were upregulated in colon cancer tissues compared to normal mucosa. Gain-of-function and loss-of-function approaches were used to examine the malignant phenotype of DPEP1-expressing or DPEP1-depleted cells. DPEP1 expression caused a significant increase in colon cancer cell adhesion and invasion *in vitro*, and metastasis *in vivo*. In contrast, DPEP1 depletion induced opposite effects. Furthermore, cilastatin, a DPEP1 inhibitor, suppressed the invasion and metastasis of DPEP1-expressing cells. DPEP1 inhibited the leukotriene D4 signaling pathway and increased the expression of E-cadherin. We also show that DPEP1 mediates TGF-β-induced EMT. TGF-β transcriptionally repressed DPEP1 expression. TGF-β treatment decreased E-cadherin expression and promoted cell invasion in DPEP1-expressing colon cancer cell lines, whereas it did not affect these parameters in DPEP1-depleted cell lines. These results suggest that DPEP1 promotes cancer metastasis by regulating E-cadherin plasticity and that it might be a potential therapeutic target for preventing the progression of colon cancer.

## INTRODUCTION

Colorectal cancer (CRC) is one of the most common neoplastic diseases in industrialized countries and is the fourth most common cause of death from cancer worldwide [1, 2]. The conversion from normal to malignant cells in CRC typically occurs over 7-12 years and involves accumulation of genetic alterations [3]. The high mortality rate of patients with CRC appears to be related to the high risk of metastasis of these cancer cells. Despite the availability of several treatment regimens for CRC, many patients die as a result of the inability to control tumor progression and metastasis [4].

Aggressive metastatic cancers are characterized by their high capacity for migration, and subsequent invasion and adhesion in distant organs [5, 6]. Acquisition of these properties by cancer cells involves aberrant changes in the expression level of several genes. Metastasis is a complex and multistep process including cancer cell detachment from the primary tumor site, local invasion to disseminate cells through surrounding lymphatic and blood vessels, and attachment and proliferation to establish secondary tumors at the metastatic sites [7]. Initial steps in metastasis are mediated by the switch of cancer cells between the epithelial and mesenchymal phenotypes, the so-called epithelial to mesenchymal transition (EMT) [8]. During cancer cell EMT, the loss of epithelial characteristics and the acquisition of a mesenchymal phenotype lead to enhanced motility and an invasive predisposition. However, metastatic cancer cells usually show heterogeneous epithelial architecture similar to that of the primary tumor [9]. Accumulating evidences have been described that epithelial-mesenchymal plasticity, referring to the reversible processes of the EMT and the mesenchymal to epithelial transition (MET), is involved in metastatic progression [10–12]. A critical event of EMT is the down-regulation of E-cadherin. Conversely, the re-expression of E-cadherin is proposed to be an important hallmark of the MET [11]. A number of EMT inducer factors have been identified, including hepatocyte growth factor, fibroblast growth factor, epidermal growth factor, and transforming growth factor-β (TGF-β). However, the factors that induce the MET are not clear. Instead, it has been proposed that a reduction of EMT inducer factors leads to the MET in distant metastatic sites [12].

Dehydropeptidase I (DPEP1), also known as membrane dipeptidase, microsomal dipeptidase, or renal dipeptidase, is a zinc-dependent metalloproteinase that hydrolyzes a variety of dipeptides and is involved in glutathione metabolism [13]. DPEP1 regulates leukotriene activity by catalyzing the conversion of leukotriene D4 (LTD_4_) to leukotriene E4 (LTE_4_) [14, 15]. Leukotrienes are known as pro-inflammatory mediators and are associated with cancer and inflammatory disease [16]. Several reports assessed the expression levels of DPEP1 mRNA in a variety of cancer cells and revealed opposite patterns depending on the tumor type. For example, a loss of DPEP1 expression is associated with Wilms's tumor and breast cancer [17, 18]. Consistent with this, DPEP1 inhibits tumor cell invasiveness and enhances chemosensitivity in pancreatic ductal adenocarcinoma [19]. In contrast, DPEP1 is highly expressed in colon tumors as compared to matched normal mucosa [20, 21]. Thus, the role of DPEP1 in tumor progression is controversial and the molecular mechanism by which it regulates tumor progression and aggressiveness remains poorly understood.

The present study was designed to uncover the putative role of DPEP1 in the progression of colon cancer. By comparing the expression profiles of DPEP1 mRNA and protein in cancer and noncancerous tissues, and characterizing the role of this gene in regulating invasion and metastasis both *in vitro* and *in vivo*, we found that DPEP1 regulates E-cadherin plasticity during TGF-β-mediated EMT, and promotes malignant progression in colon cancer.

## RESULTS

### DPEP1 expression in CRC tissues and cell lines

**Figure 1:**
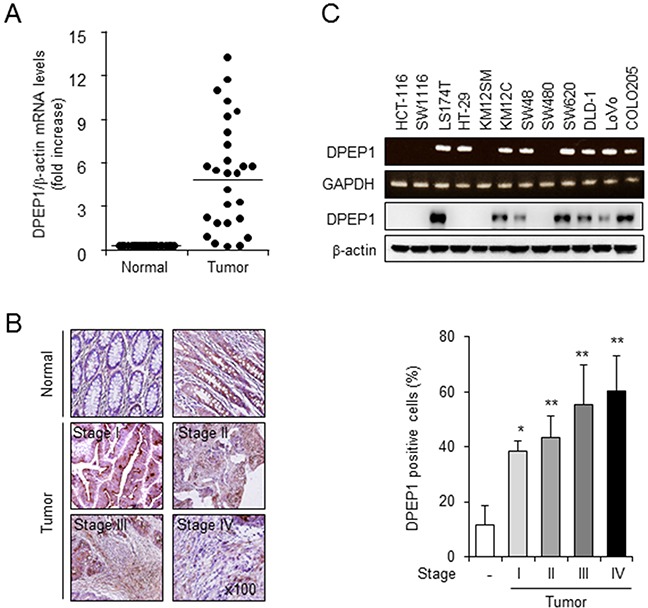
DPEP1 expression in colon cancer tissue and cell lines **A.** Real-time RT-PCR analysis of DPEP1 expression in 27 paired samples of non-tumor colon (Normal) and CRC (Tumor) tissues. β-actin was used as an internal control. **B.** Tissue array was conducted by immunohistochemistry with anti-DPEP1 antibody in normal colon (*n* = 59) and colon cancer tissues including stage I (*n* = 18), stage II (*n* = 14), stage III (*n* = 24) and stage IV (*n* = 3). Original magnification 100×. The histogram shows relative intensity of DPEP1 positive cells. The mean values and the standard error were obtained from three individual experiments. *p < 0.05, **p < 0.01. **C.** The levels of DPEP1 mRNA and proteins were detected in indicated colorectal cancer cell lines by RT-PCR and western blot analysis. GAPDH or β-actin was used as the loading control.

We previously conducted a microarray analysis with paired tumor and adjacent non-tumor tissues from 66 patients with CRC. DPEP1 was identified as one of 281 genes that were upregulated more than 2-fold in at least 60% of colorectal tumor tissues compared with normal colorectal mucosa [22]. In current study, we examined the expression levels of DPEP1 mRNA in 27 normal/tumor tissue pairs by quantitative real-time polymerase chain reaction (qRT-PCR) analysis. The level of DPEP1 mRNA averaged about 5-fold higher in colorectal tumor samples than in corresponding normal samples (Figure 1A).

To verify the expression of DPEP1 protein in CRC, we performed immunohistochemistry using a monoclonal antibody specific to this enzyme. Strong staining for DPEP1 was observed frequently in CRC tissue specimens, while negative or very weak staining was observed in normal tissue specimens (Figure 1B, left panel). Quantification of staining intensity by Image J software demonstrated that 39% of the area in tumor stage I samples, 44% in tumor stage II, 55% in tumor stage III, and 61% in tumor stage IV were DPEP1 positive, whereas 12% in normal tissues was DPEP1 positive (Figure 1B, right panel).

RT-PCR analyses showed that DPEP1 mRNA was expressed in 8 of 12 colon cancer cell lines. In addition, DPEP1 proteins were expressed in 7 colon cancer cell lines, including LS174T, KM12C, SW48, SW620, DLD-1, LoVo, and COLO205 (Figure 1C). Interestingly, DPEP1 mRNA and proteins were detected in SW620, but not SW480 cells, two cell lines derived from different stages of colon cancer in the same patient. SW480 cells were isolated from the primary tumor while SW620 cells were isolated from a lymph node metastasis [23]. Together, these finding led us to hypothesize that DPEP1 expression may influence the malignant progression of human colon cancer cells.

### DPEP1 promotes colon cancer cell invasion and adhesion

**Figure 2:**
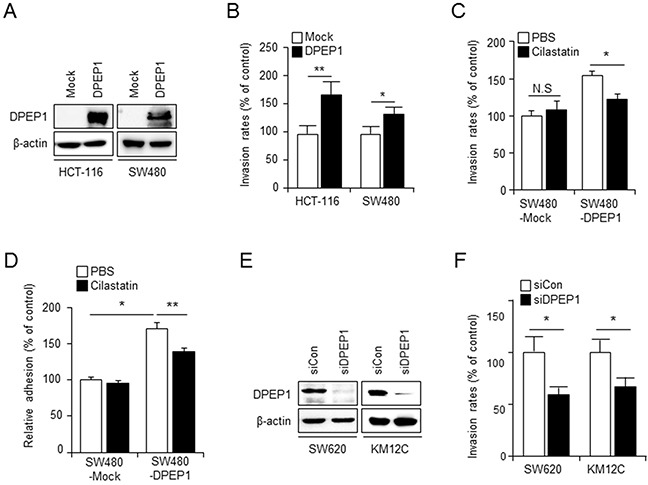
DPEP1 promotes colon cancer cell invasion and adhesion **A.** HCT-116 and SW480-derived cell lines were stably transfected with empty (Mock) or DPEP1-expressing vector (DPEP1). DPEP1 expression was analysed by immunoblotting. **B.** Effect of DPEP1 overexpression on invasion ability of HCT-116 and SW480 cells. The invasion activity of each clone was measured with modified Boyden chambers as described under “Materials and Methods”. **C.** Effect of DPEP1 inhibitor on invasiveness of SW480-Mock or SW480-DPEP1 cells. Cells were treated with PBS (vehicle control) or 100 μg/ml of cilastatin sodium and the invasion activity was measured **D.** SW480-Mock or SW480-DPEP1 cells were pre-treated with PBS or 100 μg/ml of cilastatin sodium for 1 h and then re-plated in the matrigel coated plates for 2 h. Adhesion assay were performed as described under “Materials and Methods”. **E.** SW620 or KM12C cells were transfected with control (siCon) or DPEP1 (siDPEP1) siRNA. 48 h after transfection, DPEP1 expression was analysed by immunoblotting. **F.** Effect of DPEP1 depletion on invasion ability of SW620 and KM12C cells. The mean values and the standard error were obtained from three individual experiments. *p < 0.05, **p < 0.01.

In an effort to determine whether DPEP1 expression was associated with the metastatic ability of CRC cells, we established DPEP1-overexpressing cell lines from HCT-116 and SW480 cell lines that do not express endogenous DPEP1 (Figure 2A). Both HCT-116 and SW480 cells expressing DPEP1 showed an increased invasive ability compared to the corresponding vector-transfected control cells (Figure 2B). Both cell lines exhibited similar growth rates (data not shown), thereby indicating that an increase of tumor cell invasiveness via the expression of DPEP1 was not associated with proliferation.

To test whether DPEP1 activity was necessary for invasiveness, SW480-mock or SW480-DPEP1 cells were treated with the selective DPEP1 inhibitor cilastatin sodium. Cilastatin had no effect on the invasion of SW480-mock cells, but significantly prevented the invasion of SW480-DPEP1 cells (Figure 2C). This indicated that DPEP1 activity was involved in invasion.

Because the binding of cells to the extracellular matrix plays a significant role during metastasis [5], we plated SW480-mock or SW480-DPEP1 cells on matrigel-coated plates and conducted an adhesion assay. SW480-DPEP1 cells showed increased adhesion ability compared to control SW480-mock cells. In addition, cilastatin attenuated DPEP1-mediated cell adhesion (Figure 2D).

The effects of DPEP1 on tumor cell invasion were assessed further by transfecting SW620 and KM12C cells with control or DPEP1-specific siRNA. Western blot analyses (Figure 2E) showed that almost no DPEP1 could be detected in SW620 and KM12C cells transfected with DPEP1 siRNA. DPEP1 depletion significantly reduced the invasiveness of each cell line (Figure 2F). These data suggest that DPEP1 promotes cell adhesion and invasion and that dipeptidase activity is involved in this function.

### DPEP1 increases E-cadherin expression through inhibition of the LTD_4_ signaling pathway

**Figure 3:**
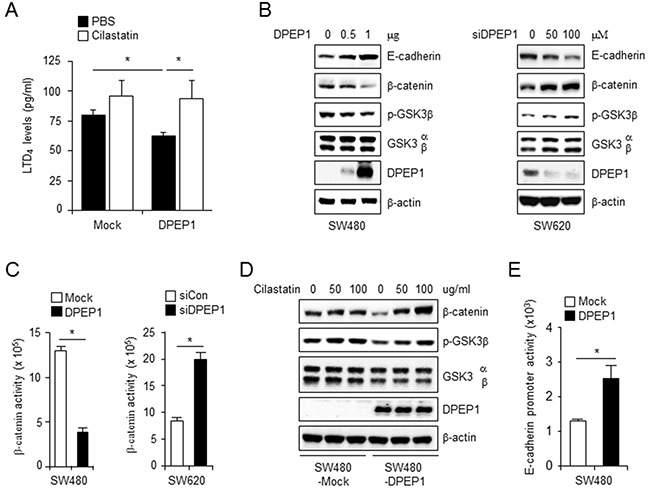
DPEP1 increases E-cadherin expression through inhibition of LTD4 signaling pathway **A.** SW480-Mock and SW480-DPEP1 cells were treated with PBS or 100 μg/ml cilastatin sodium and LTD_4_ levels were measured as described under “Materials and Methods”. **B, C.** SW480 or SW620 cells were transfected with DPEP1 expressing vector or DPEP1 siRNA at indicated concentrations, respectively. (B) Cell lysates were analysed by immunoblotting with indicated antibodies. (C) β-catenin activity was measured by TopFlash assay as described under “Materials and Methods”. **D.** SW480-Mock or SW480-DPEP1 cells were treated with cilastatin sodium at indicated concentrations. Cell lysates were analysed by immunoblotting with indicated antibodies. **E.** SW480 cells were transfected with mock or DPEP1 expressing vector for 24 h. E-cadherin promoter activity were measured by luciferase assay as described under “Materials and Methods”. The mean values and the standard error were obtained from three individual experiments. *p < 0.05.

Because DPEP1 regulates leukotriene activity by catalyzing the conversion of LTD_4_ to LTE_4_ [14, 15], we hypothesized that DPEP1 regulates LTD_4_ signaling. First, we assessed whether DPEP1 reduced LTD_4_ concentrations using a LTD_4_ ELISA kit. SW480 cells expressing DPEP1 showed reduced LTD_4_ levels compared to control SW480-mock cells. Cilastatin blocked this suppressive effect (Figure 3A).

Signaling through LTD_4_ and its cognate receptor, CysLT1R, increases the level of β-catenin through the inhibition of glycogen synthase kinase-3β (GSK-3β) activity and subsequent decreases in E-cadherin expression [24]. Phosphorylation at tyrosine 216 of GSK-3β is required for its kinase activity. In contrast, phosphorylation at serine 9 inhibits GSK-3β activity [25]. Therefore, we next determined whether DPEP1 regulates the levels of β-catenin and GSK-3β phosphorylation. Western blot analysis showed that overexpressing DPEP1 in SW480 cells caused a decrease in phosphorylation at ser-9 of GSK-3β (an increase in GSK-3β activity) and a reduction in β-catenin. In contrast, depleting DPEP1 in SW620 cells resulted in increased GSK-3β phosphorylation and increased β-catenin levels (Figure 3B).

The effect of DPEP1 on β-catenin activity was confirmed with the TopFlash (TCL/LEF-Firefly luciferase) assay. Consistent with the western blot analysis, β-catenin activity was lower in DPEP1-expressing SW480 cells and higher in DPEP1-depleted SW620 cells, as compared to its activity in control cells (Figure 3C). Treatment of DPEP1-expressing SW480 cells with cilastatin inhibited the DPEP1-mediated decrease in GSK-3β phosphorylation and restored β-catenin expression in a concentration-dependent manner, but had no significant effect in control SW480 cells (Figure 3D).

Because LTD_4_ downregulates E-cadherin expression through the nuclear translocation and activation of β-catenin [24], we next verified the effect of DPEP1 on E-cadherin expression. As expected, western blot analyses and the luciferase assay showed that overexpressing DPEP1 in SW480 cells significantly increased E-cadherin protein levels (Figure 3B) and promoter activity (Figure 3E). In addition, depleting DPEP1 in SW620 cells by siRNA reduced the expression of E-cadherin (Figure 3B). Collectively, these results indicate that DPEP1 inhibits the LTD_4_ signaling pathway through the conversion of LTD_4_ to LTE_4_ and results in an increase in E-cadherin expression.

### DPEP1 regulates E-cadherin plasticity in TGF-β-mediated EMT

**Figure 4:**
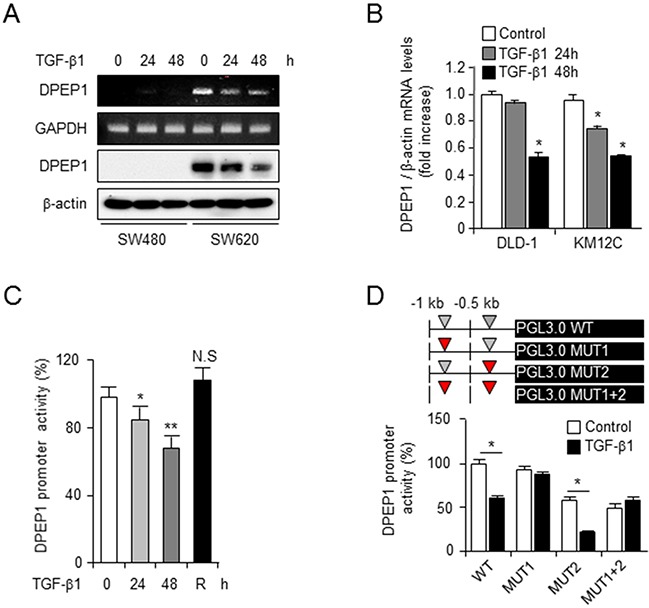
TGF-β transcriptionally suppresses the expression of DPEP1 **A.** SW480 or SW620 cells were treated with 10 ng/ml of TGF-β1 for indicated times. The levels of DPEP1 expression was analysed by RT-PCR or immunoblotting. GAPDH and β-actin were used for loading control. **B.** DLD1 or KM12C cells were treated with 10 ng/ml of TGF-β1 for indicated times. The levels of DPEP1 mRNA was analysed by real time RT-PCR. **C.** SW480 cells were transfected with PGL3 luciferase vector containing DPEP1 promoter and treated with 10 ng/ml of TGF-β1 for indicated times. 48 h after treatment with TGF-β1, the recovery experiment (R) was performed by removing TGF-β1 and incubating for additional 24 h. **D.** SW480 cells were transfected with wild-type or mutants (MUT1, MUT2, or MUT1+2) of DPEP1 promoter and treated with 10 ng/ml of TGF-β1 for 48 h. DPEP1 promoter activity was analysed by luciferase assay as described in “Materials and Methods”. The mean values and the standard error were obtained from three individual experiments. *p < 0.05, **p < 0.01.

Phenotypic switching between epithelial- and mesenchymal-type cells is essential for local invasion and distant metastasis. TGF-β is the core EMT transcription factor and plays important roles in the EMT/MET switch [26]. To investigate the mechanism regulating DPEP1 expression in colon cancer, we examined the levels of DPEP1 mRNA and protein in the presence of TGF-β1 in the SW480 and SW620 colon cancer cell lines. RT-PCR and western blot analyses showed that DPEP1 expression was significantly reduced by treatment with TGF-β1 in SW620 cells that express endogenous DPEP1 (Figure 4A). However, TGF-β1 did not affect DPEP1 expression in SW480 cells that do not express endogenous DPEP1 (Figure 4A). We confirmed the effect of TGF-β1 on DPEP1 mRNA expression in two other cell lines, DLD1 and KM12C that express endogenous DPEP1. Treatment with TGF-β1 attenuated DPEP1 mRNA expression in both lines in a time-dependent manner (Figure 4B).

To estimate DPEP1 promoter activity in response to TGF-β1, SW480 cells were transfected with a PGL3-luciferase reporter vector containing -1kb of the DPEP1 promoter. These cells were then treated with TGF-β1 for up to 48 h. Luciferase promoter activity was significantly decreased by TGF-β1 in a time-dependent manner. When TGF-β1 was removed and the cells were incubated for an additional 24 h, luciferase activity recovered (Figure 4C). Because there are two putative Smad binding element (SBE) core sequence CAGACA in the DPEP1 promoter (SBE1; -802/-808 and SBE2; -255/-261), we constructed three mutants (CAGACA>ACGCGT), MUT1, MUT2, and MUT1+2 (Figure 4D). The luciferase assay was performed with the PGL3-luciferase vector containing the wild type, MUT1, MUT2, or MUT1+2 promoter. Wild type and MUT2 promoter activity significantly decreased in the presence of TGF-β1. In contrast, TGF-β1 did not affect MUT1 and MUT1+2 promoter activity (Figure 4D). This suggests that the Smad binding element 1 region in the DPEP1 promoter is essential for the inhibition of DPEP1 expression by TGF-β1.

**Figure 5:**
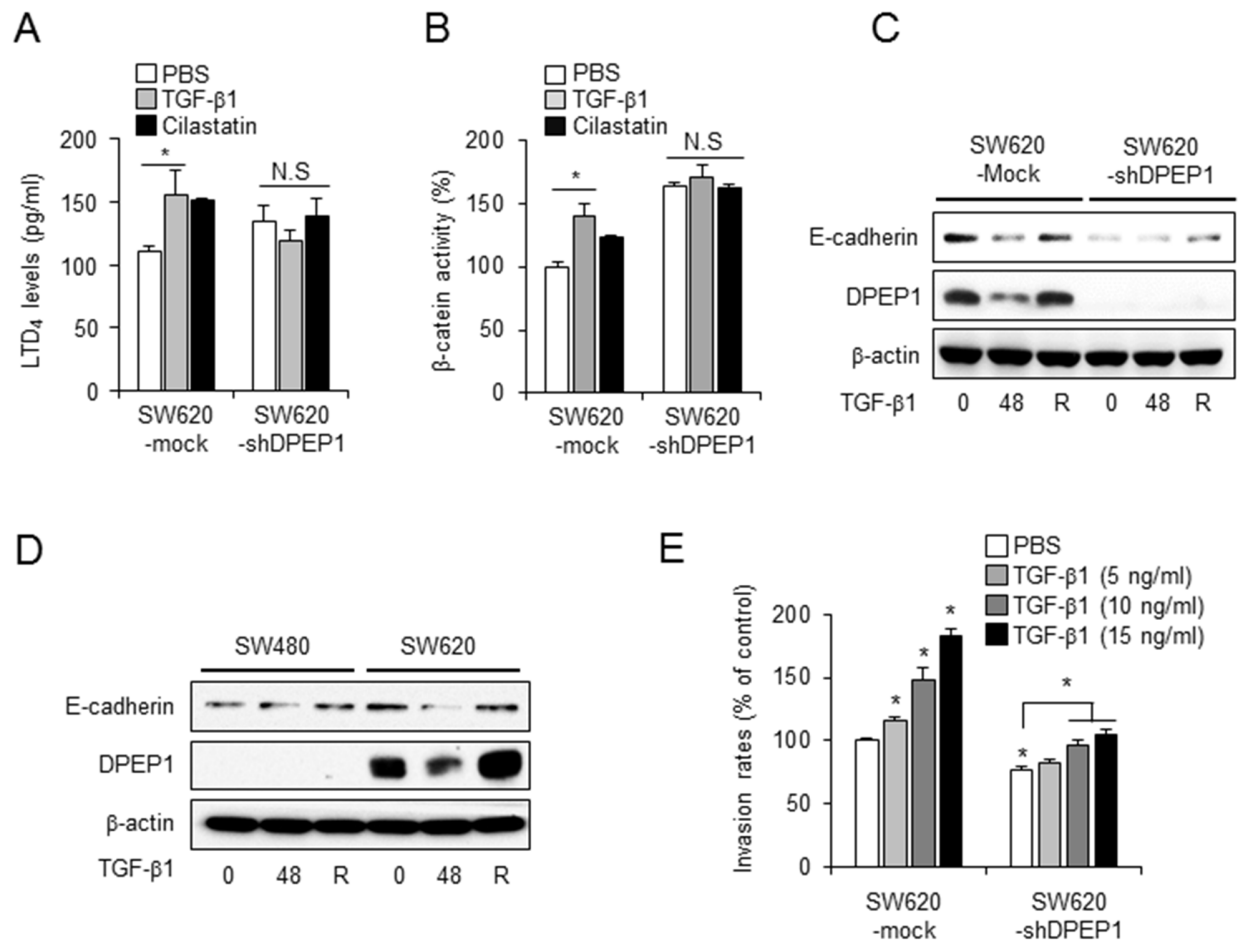
DPEP1 regulates E-cadherin plasticity in TGF-β-mediated EMT SW620 cells stably transfected with control (SW620-mock) or DPEP1 shRNA (SW620-shDPEP1) were treated with 10 ng/ml of TGF-β1 or 100 μg/ml of cilastatin sodium for 48 h. LTD_4_ levels **A.** and β-catenin activity **B.** were analysed by LTD4 ELISA or TopFlash assay, respectively. **C.** SW620-Mock or SW620-shDPEP1 cells were treated with TGF-β1 for 48 h. **D.** SW480 or SW620 cells were treated with TGF-β1 for 48 h. The recovery was performed by removing TGF-β and incubating for additional 24 h. The expression levels of DPEP1 and E-cadherin were analysed by immunoblotting using indicated antibodies. β-actin were used for loading control. **E.** SW620-Mock or SW620-shDPEP1 cells treated with PBS (vehicle control) or TGF-β1 for 48 h at indicated concentrations. Invasion assay were performed as described under “Materials and Methods”. The mean values and the standard error were obtained from three individual experiments. *p < 0.05.

Because DPEP1 downregulates LTD_4_ levels and β-catenin activity, and upregulates E-cadherin expression, we evaluated whether TGF-β1 affected the DPEP1 downstream signaling pathway. The LTD_4_ ELISA and TopFlash assay showed that the LTD_4_ level and β-catenin activity were increased by treatment with TGF-β1, as well as by a DPEP1 inhibitor, in SW620 mock cells. In contrast, there were no changes in these parameters in DPEP1-depleted SW620 cells, although the activity in the control DPEP1-depleted cells was higher than that in mock cells (Figures 5A and 5B).

We next investigated whether DPEP1 was required for the TGF-β1-mediated E-cadherin repression. Consistent with the DPEP1 promoter luciferase assay (Figure 4C), the expression of DPEP1 protein was reduced by stimulation with TGF-β1 and recovered following its removal in both SW620 and SW620-mock cells that express endogenous DPEP1 (Figure 5C and 5D). Interestingly, E-cadherin expression was also effectively downregulated by TGF-β1 and restored by its removal in SW620 and SW620-mock cells. In contrast, we detected no difference in the expression of E-cadherin in SW480 or DPEP1-depleted SW620 cells (Figure 5C and 5D), suggesting that TGF-β1 activates the LTD_4_/β-catenin pathway and decreases E-cadherin expression through the inhibition of DPEP1 expression.

We next evaluated the TGF-β1-mediated invasion activity of SW620-mock and DPEP1-depleted SW620 cells. Consistent with the findings shown in Figure 2F, SW620-mock cells showed a higher invasion ability than DPEP1-depleted SW620 cells. Moreover, when cells were stimulated with TGF-β1, the invasion activity of SW620-mock cells was significantly enhanced in a concentration dependent manner, whereas DPEP1-depleted SW620 cells showed only a slight increase (Figure 5E). Collectively, these data suggest that DPEP1 mediates E-cadherin repression and cell invasion in response to TGF-β1.

### DPEP1 promotes colon cancer metastasis in xenograft mice

**Figure 6:**
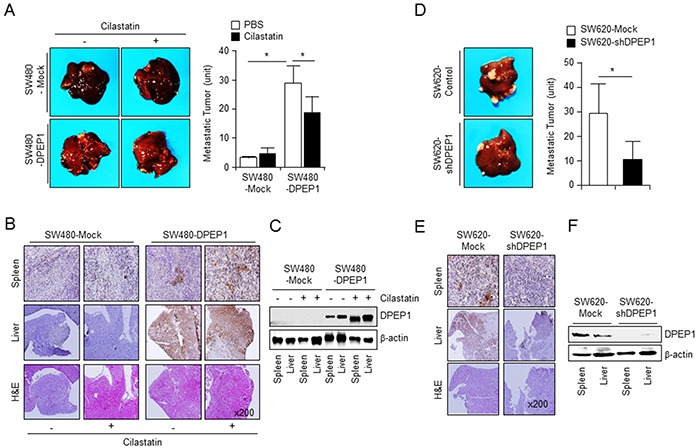
DPEP1 increases metastatic activity of colon cancer cells *in vivo* **A.** SW480-Mock (*n* = 6) or SW480-DPEP1 (*n* = 6) cells were injected into spleen of 6-week-old nude mice. The mice were treated with PBS or cilastatin sodium (10 mg/kg or mice, 3 times a week) by tail vein injection. The number of metastatic liver nodules in individual mice was counted under the microscope. DPEP1 expression was analysed by immunohistochemistry **B.** and Western blot analysis **C. D.** SW620-Mock or SW620-shDPEP1 cells were injected into spleen of 6-week-old nude mice. The mice were treated with PBS or cilastatin sodium. The number of metastatic liver nodules in individual mice was counted under the microscope. DPEP1 expression was analysed by immunohistochemistry **E.** and Western blot analysis **F.** The mean values and the standard error were obtained from three individual experiments. *p < 0.05.

Intrasplenic injection of colon cancer cells is an effective method of developing liver metastasis in nude mice [27]. SW480-Mock or SW480-DPEP1 cells were injected into the spleen of nude mice. Mice were then treated with phosphate-buffered saline or cilastatin at intervals of 2 days by tail vein injection. After 4 weeks, mice were euthanized and the number of metastatic nodules located at the liver surface was counted. Numerous liver metastatic nodules were observed in mice injected with DPEP1-expressing SW480 cells. In contrast, only a few nodules were detected in mice injected with SW480-mock cells. Moreover, liver metastatic nodules of SW480-DPEP1 cells were reduced by cilastatin, but those of SW480-mock cells were not (Figure 6A). The expression of DPEP1 in spleen and liver tissue sections was confirmed by immunostaining and western blot analyses. DPEP1 was detected in spleen and liver tissues of mice injected with SW480-DPEP1 cells but not in mice injected with SW480-mock cells (Figure 6B and 6C).

To further assess liver metastasis, we injected SW620-mock or SW620-shDPEP1 cells into the spleen of nude mice. There were a large number of metastatic liver nodules in mice injected with control SW620-mock cells, whereas there were a significantly decreased number of nodules in mice injected with DPEP1-depleted SW620 cells (Figure 6D). Immunostaining and western blot analyses showed that DPEP1 was expressed in spleen and liver tissues of mice injected with SW620-mock cells but not in mice injected with SW620-shDPEP1 cells (Figure 6E and 6F). These results demonstrate that DPEP1 plays a crucial role in colon cancer cell metastasis.

## DISCUSSION

DPEP1 is a zinc-dependent metalloproteinase that hydrolyses a variety of dipeptides and is involved in glutathione metabolism [13, 28]. This protein has a highly hydrophobic sequence at its carboxyl terminus and is anchored to the membrane through a covalent attachment to glycosyl phosphatidylinositol [29]. DPEP1 was identified as a tumor suppressor due to its decreased expression in Wilms’ tumor as compared to normal kidney tissue [17]. Similarly, loss of DPEP1 expression correlates with breast lobular carcinomas [18]. DPEP1 expression is also negatively associated with the histological grade of pancreatic ductal adenocarcinoma, and its overexpression suppresses tumor cell invasiveness and enhances chemosensitivity of this cancer [19].

The results of the current study provide strong evidence that DPEP1 functions as a positive regulator for metastasis in colon cancers. This discrepancy from previous studies suggests there are different roles for DPEP1 in the progression of colon cancer compared with other cancers. We found that the expression levels of DPEP1 mRNA and proteins were much higher in CRC tissues than normal mucosa. In addition, immunohistochemical results showed that the expression level of DPEP1 increased at higher colorectal tumor stages (Figure 1). We also found that the enhanced expression of DPEP1 increases cancer cell invasiveness, whereas its depletion by RNA interference causes the opposite effect (Figure 2). In addition, overexpression of DPEP1 in SW480 cells significantly enhances liver metastasis, whereas knockdown of DPEP1 in SW620 cells reduces liver metastasis in a xenograft model (Figure 6). Finally, a pharmacological inhibitor of DPEP1 suppresses cancer cell invasion *in vitro* and liver metastasis *in vivo* (Figures 2 and 6). Consistent with our findings, some reports show that DPEP1 mRNA is highly expressed in colon tumors compared to matched normal mucosa, and its expression is associated with the histological stage of colon cancer [20, 21].

Although accumulating evidence suggests that DPEP1 is involved in cancers, the mechanisms by which this enzyme inhibits or promotes tumor progression and aggressiveness are not known. In the current study, we provide evidence regarding the molecular mechanism by which DPEP1 regulates colon cancer metastasis. DPEP1 affects leukotriene activity by promoting the conversion of LTD_4_ to LTE_4_ [15]. Accordingly, DPEP1 overexpression reduces the concentration of LTD_4_ in the cell culture medium and suppresses LTD_4_-mediated downstream signaling, including inhibition of GSK3-β and activation of β-catenin. In contrast, depleting DPEP1 causes the opposite effects. Inhibition of LTD_4_ signaling by DPEP1 enhances E-cadherin expression (Figure 3). This is an unexpected result because the EMT is associated with enhanced cell migration and invasion and requires disruption of apical-basal polarity and loss of E-cadherin expression. Because DPEP1 increased colon cancer cell invasion, we expected that the expression of E-cadherin would be decreased by DPEP1. Interestingly, there is mounting evidence of high expression or re-expression of E-cadherin in advanced metastatic tumors [30, 31]. Indeed, the E-cadherin-positive prostate tumor stem cell population is highly invasive and capable of altering its E-cadherin expression during invasion [32]. In addition, some reports suggest that lymphatic metastasis involves collective cell migration associated with a more epithelial phenotype, whereas vascular invasion involves amoeboid motility with the mesenchymal phenotype [33, 34]. Our data support the importance of an epithelial phenotype for cancer cell invasion and metastasis.

EMT is induced by some growth factors such as epidermal growth factor, hepatocyte growth factor, and TGF-β. However, the factor inducing the MET is unknown. Instead, it is believed that reduction in EMT inducer factors reverses the EMT at distant metastatic sites [10]. TGF-β functions in tumor progression as both a tumor suppressor and promoter. In the normal epithelium, TGF-β appears to be a tumor suppressor due to its ability to inhibit proliferation and induce apoptosis. However, TGF-β promotes tumor progression and induces a more aggressive phenotype in malignant tumors [26, 35]. In the current study, TGF-β transcriptionally suppressed the expression of DPEP1 (Figure 4). Stimulation of DPEP1-expressing cells with TGF-β1 downregulated E-cadherin and significantly increased cell invasion. In addition, E-cadherin was restored after removing TGF-β1 in DPEP1 expressing cells. However, TGF-β1 did not affect E-cadherin expression and slightly increased cell invasion in DPEP1 non-expressing cells (Figure 5). Therefore, these data indicate that DPEP1 is critical for TGF-β-mediated E-cadherin repression and at least partially mediates cell invasion in response to TGF-β in colon cancer cells. Although the molecular mechanism by which DPEP1 regulates the TGF-β response remains unclear, in agreement with our concept, highly invasive and metastatic side population pancreatic cancer cells show increased E-cadherin expression and TGF-β responsiveness on E-cadherin plasticity and invasion, compared to control cells [36].

Despite advances in our understanding of colon cancer at the molecular level and the emergence of targeted therapy for this disease, predictive or therapeutic biomarkers remain elusive. The present study has revealed DPEP1 as a mediator for colon cancer progression that promotes cancer cell invasion and metastasis by regulating E-cadherin plasticity. These findings provide new insights into the molecular mechanisms underlying DPEP1-induced colon cancer. We propose that DPEP1 could be a potential therapeutic target as well as a prognostic marker for colon cancer.

## MATERIALS AND METHODS

### Cell culture

The colorectal cancer cell lines HCT-116, SW1116, LS174T, HT-29, KM12C, KM12SM, SW48, SW480, SW620, DLD-1, LOVO, COLO205 were obtained from the Korean Cell Line Bank (Seoul, Korea) and were maintained in DMEM (Gibco-BRL, Grand Island, NY) supplemented with 10% fetal bovine serum and 100 μg/ml antibiotics (100 U/ml penicillin and 100 μg/ml streptomycin. Cells were maintained at 37°C in a humidified, 5% CO_2_/air atmosphere.

### Construction of the DPEP1 expression plasmid and transfection

Human DPEP1 cDNA (NM_001128141.2) was amplified by PCR. PCR products were cloned in to the EcoR1/Sal1 site of pEGFPN2 vector. Transfection was performed using the Lipofectamine 2000 reagent (Invitrogen, Carlsbad, CA, USA) according to the instructions of the manufacturer. After 24 h of incubation, cells were selected by culturing in the presence of G418 (Calbiochem, Billerica, MA, USA).

### RNA interference experiments

DPEP1-specific siRNA and control siRNA were purchased from Bioneer (Daejeon, korea). The sequence of DPEP1 siRNA was 5′-CAG UUC UGG UCC GUG UAC AdTdT-3′. Colorectal cancer cells were transfected with DPEP1 siRNA or control siRNA using Lipofectamine 2000 reagent. The short hairpin RNA (shRNA) targeting DPEP1 was obtained from SIGMA (TRCN0000046648, St. Louis MO, USA). shRNA expression vector was transfected into lentiviral packaging cell lines 293T cells. The culture supernatant containing virus particles was harvested 48 h after transfection. For stable transduction of lentivirus, cells at 60% to 70% confluency were grown in six-well plates, and 1 ml of viral supernatant containing 4 μg/ml of polybrene was added. After 48 h, 1 μg/ml puromycin (Clontech, Mountain View, CA, USA) was added to the cultures for selection.

### Quantitative real time PCR

Total RNA was prepared using Trizol RNA Isolation Reagents (Invitrogen, Carlsbad, CA, USA) according to the manufacturer's instruction. Reverse transcription was conducted using 10 μg of total RNA with a reverse transcription kit (Promega, Madison, WI, USA). 1 ml of cDNA was used for the PCR, and triplicate reactions were performed for each sample using a Power SYBR Green Kit (Applied Biosystems, Foster City, CA, USA) with gene-specific primers on an ABI StepOnePlus instrument. The PCR primers were as follows: DPEP1, 5′-ACTTGGCTCACGTGTCTGTG-3′ (sense) and 5′-TGTCTGTTTCACCAGCCTCA-3′ (antisense); E-cadherin, 5′-GAACGCATTGCCACATACAC-3′ (sense) and 5′-GAATTCGGGCTTGTTGTCAT-3′ (antisense); β-actin, 5′-AAGGCCAAC CGCGAGAAGAT-3′ (sense) and 5′-TGATGACCTGGCCGTCAGG-3′ (antisense). RNA quantity was normalized to β-actin content, and gene expression was quantified according to the 2^−ΔCt^ method.

### Western blot analysis

Cells were lysed in RIPA lysis buffer (50 mM Tris-HCl (pH 7.4), 150 mM NaCl, 1% NP40, 0.25% sodium deoxycholate, 1 mM phenylmethylsulfonylfluoride, protease inhibitor mixture (Sigma, St. Louis MO, USA), 1 mM sodium orthovanadate). Equivalent amounts of protein lysate were separated by SDS-PAGE, transferred to PVDF membrane and incubated with the followed primary antibodies: β-actin, E-cadherin, GFP, β-catenin, GSK3-αβ (Santa Cruz Biotechnology, Santa Cruz, CA, USA), p-GSK3β (Cell Signaling, Danvers, MA, USA), DPEP1(Sigma, St. Louis MO, USA). The bound antibodies were visualized with a suitable secondary antibody conjugated with horseradish peroxidase using enhanced chemiluminescence (Amersham Bioscience, Pittsburgh, PA, USA).

### Measurement of cysteinyl leukotrienes D4

Cells were seeded into 24 well culture plates at 2 × 10^5^ cells/well in 0.5 mL standard culture medium for 24 h. Culture supernatants were harvested 48 h after serum starvation. The measurement of LTD_4_ was performed using the leukotriene D_4_ ELISA kit (Mybiosource, San Diego, CA, USA) according to the manufacturer's instruction. Absorbance was measured at 450 nm using a microplate reader.

### Adhesion assay

96 well culture plate were pre-coated with matrigel (BD Bioscience, Franklin Lakes, NJ, USA), followed by blocking with 1% BSA. Cells were treated with PBS or 100 μg/ml of cilastatin sodium for 1 h. 2 × 10^4^ cells were re-plated in the matrigel coated 96 well plates and allowed to adhere for 2 h. Cells were then gently washed with PBS. After washing, remained adhesion cells were stained with 0.1% crystal violet for 30 min and distained with 10% acetic acid for 15 min. Absorbance was measured at 595 nm using a microplate reader.

### Invasion assay

The invasion ability of cancer cells were assessed using a matrigel-based transwell system. Briefly, 24 well culture plate inserts with 8-μm pore size polycarbonate membrane (Corning, Corning, NY, USA) were pre-coated with 100 μl matrigel (BD Bioscience, Franklin Lakes, NJ, USA). 2 × 10^5^ cells in 200 μl of serum-free media were placed in the insert and the lower chamber was filled with 600 μl of DMEM containing 10% FBS, and allowed to invade for 48 h. After incubation, non-invading cells on upper surface of the insert were removed with cotton swab. Invaded cell were stained with crystal violet. Absorbance was measured at 595 nm using a microplate reader

### Luciferase reporter assay

Cells were seeded in 24 well plates and transfected with PGL3-firefly luciferase vector containing wild-type or mutant DPEP1 promoter. To evaluate the E-cadherin promoter activity, cells were transfected with a reporter luciferase vector containing E-cadherin promoter (−368 ∼ +51). Luciferase activity was quantified 48 h after transfection using the Dual-Luciferase Reporter assay system (Promega, Madison, WI, USA), following the manufacturer's instruction. The luciferase activity was normalized to activity of Renilla luciferase. The data represented at least three independent experiments.

### Immunohistochemistry

A human colon cancer tissue array (59 cases; CDA3) was purchased from SuperBioChips (Seoul, Korea). Cancer tissues with corresponding normal tissue slides were deparaffinized by xylen, and antigen retrieval was carried out in citrate buffer for 10 min. The slides were washed in PBS and incubated in blocking solution. The slides were immunohistochemically stained with diluted primary antibody against DPEP1 (1:100) using DAB substrate kit (Vector laboratories, Burlingame, CA, USA). Relative intensity of DPEP1 were quantified using image J software (http://openwetware.org/wiki/Sean_Lauber:ImageJ_-_Threshold_Analysis). The % stained area is determined as the IHC stained area (brown staining)/total area (brown + non-brown staining) × 100.

### Animal models

Female 6 week-old BALB/c nude mice were purchased form Central Lab, Animal Inc (Seoul, Korea). Mice were housed in a pathogen-free condition room in Animal Care Facility at the Korea Research Institute of Bioscience and Biotechnology (KRIBB). All experiments were performed following the Animal Care and Use guidelines of the Korea Research Institute of Bioscience and Biotechnology (Daejeon, Korea). For the xenograft assay, SW480-Mock, SW480-DPEP1, SW620-shCon and SW620-shDPEP1 cells (2 × 10^6^ cells/mouse in 50ul PBS) were injected into spleen. The mice were treated with PBS or cilastatin sodium (10 mg/kg or mice, 3 times a week) by tail vein injection. 4 weeks after cell injection, liver metastasis was quantified by counting metastatic nodules.

### Statistical analysis

The Student's *t* test was used to evaluate experimental data. Values with *P* < 0.05 were considered to be statistically significant.
